# Technical Perspective of Landfill Leachate Treatment via Membrane Distillation Using Waste Heat from Biogas Combustion for Power Generation

**DOI:** 10.1021/acsomega.6c01204

**Published:** 2026-07-10

**Authors:** Bruno V. M. da Silva, Fabiana V. da Fonseca, Cristiano P. Borges

**Affiliations:** † Escola de Química, 28125 Universidade Federal do Rio de Janeiro, 21945-970 Rio de Janeiro, Brazil; ‡ Programa de Engenharia Química, COPPE, Universidade Federal do Rio de Janeiro, 21941095 Rio de Janeiro, Brazil

## Abstract

In this study, we investigated the potential of using the combustion of biogas generated in landfills for power generation as an alternative waste heat resource for landfill leachate treatment. The effectiveness of direct-contact membrane distillation (DCMD) for wastewater treatment was also evaluated. The performance of DCMD was assessed based on conductivity, chemical oxygen demand (COD), and ammonia retention by testing both synthetic and raw landfill leachate. Results showed that the permeate stream had low COD (<0.5 mg/L) and turbidity (<1 NTU); however, ammoniacal nitrogen exceeded the maximum discharge limits defined by Brazilian legislation, indicating the need for additional treatment. Energy consumption analysis through simulations demonstrated that partial feed recycling in DCMD improved energy efficiency, achieving a gain output ratio (GOR) of 2.54 and reducing specific energy consumption (SEC) by 25%. The simulations showed that landfills producing more than 6.4 m^3^/h landfill leachate can meet treatment demands with on-site energy, while smaller facilities may require supplemental energy. Full allocation of biogas energy enables complete water recovery at all sites, demonstrating the feasibility of DCMD in landfill operations.

## Graphical abstract

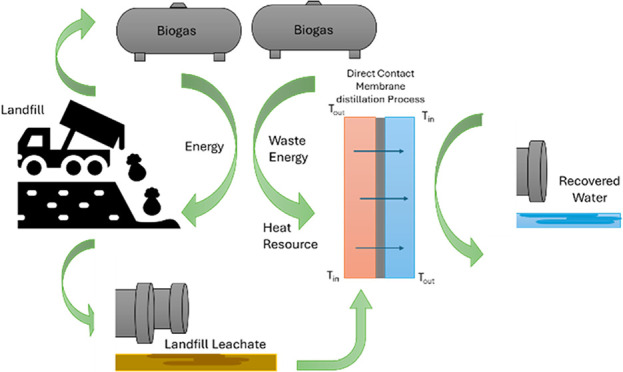

## 1 Introduction

Worldwide, human activities have generated waste. Over the years, waste generated by the population has been increasing and has become a severe problem in many countries because of poor management. The absence or inadequate management of municipal solid waste (MSW) leads to various environmental problems, such as contamination of water, soil, and the atmosphere.^1^ Currently, MSW disposal in sanitary landfills is the most common method of solid waste management in developing and undeveloped countries. However, strict surveillance of the project and operation of landfills is essential because of the leachate generated, which can contaminate nearby groundwater or surface water.^2^

Landfill leachate is a highly variable heterogeneous wastewater generated in landfills that may contain many inorganic and organic compounds. Landfill leachate contributes to rainwater percolation through the layers of waste by dissolving and transporting the matter inherent to the residues themselves or resulting from their decomposition.^2–4^

Due to the complexity of the leachate, on-site treatments usually consist of implementing a multistage approach based on physical/chemical/biological techniques such as membrane bioreactor (MBR) and nanofiltration (NF); semiaerobic aged refuse biofilter (SAARB) and ozonation processes;^5^ aerobic biological treatment, ultrafiltration (UF), and ion-exchange processes;^6^ coagulation and ionization processes;^7^ nitrification–denitrification, coagulation/flocculation, and different advanced oxidation (AOP) processes;^8^ and anaerobic digestion, lime precipitation, microfiltration (MF), and reverse osmosis (RO).^9^ The choice of an ideal route of treatment technique depends on leachate characteristics and process viability, such as the cost and available area.

Membrane distillation (MD) is an emerging thermally driven membrane technology, and its application has been attracting attention not only for desalination processes^10^ but also for several wastewater treatments.^11–14^ The process consists of thermally driven transport of vapor through a nonwetted porous hydrophobic membrane; the driving force is the vapor pressure difference between the two sides of the porous membrane.^15,16^ MD is becoming a promising separation technique owing to the benefits of the process, such as the low temperature and low transmembrane hydrostatic pressure required to perform its operation, and the possibility of using renewable energies such as solar^17–19^ and geothermal energy^20–22^ or waste heat^23–26^ as heat sources.

The treatment of landfill leachate by membrane distillation has recently been studied. Nghiem, Hai, and Listowski^27^ were the first to apply MD to a combined process for landfill leachate treatment. The authors noted that the combination of electrocoagulation, NF, and MD was effective and produced high-quality water for reuse. Zhou et al.^28^ combined the MD process with forward osmosis (FO) and achieved a rejection of over 98% of total organic carbon (TOC) and total nitrogen (TN) and complete removal of NH_4_^+^-N, Hg, and Sb. Zhang et al.^29^ investigated the scaling phenomena in FO-MD processes and identified through long-term experiments that the integrated process was much better than FO alone, and the application of an organic phosphonic acid scale inhibitor not only helped prevent the scaling phenomena in FO but also avoided the wetting of the MD membrane. Zoungrana et al.^30^ applied DCMD to investigate the treatability of raw and pretreated landfill leachate. The authors identified high process efficiency in pretreated landfill leachate compared to raw landfill leachate, such as the rejection of COD (99% and 98%) and NH_4_^+^-N (92 and 70%), and in transmembrane fluxes (15.54 and 9.87 L/m^2^ h). Yan et al.^31^ evaluated the application of MD in landfill leachate treatment and the mechanisms of membrane fouling during this process. The authors achieved high rejection of TOC (>98.93%), phosphate (>99.19%), and metal ions (99.95%). Regarding the fouling mechanism, the authors detected an increase in the phenomenon at an alkaline pH.

Ammonia recovery from landfill leachate has also been studied with the application of an MD process. Zoungrana et al.^30^ identified that pH, temperature, and salt concentration highly affect the efficiency of the NH_3_ recovery. The authors also observed that the ammonia recovery in synthetic landfill leachate was higher than that in raw landfill leachate, with 96.6 and 58.45% yields, respectively. Zico et al.^32^ applied solar-driven modified direct-contact membrane distillation to achieve 98% ammonia removal and 59% ammonia recovery from landfill leachates.

In addition, the application of waste energy for MD processes has recently become the focus of some research. MD technology is widely recognized as energy-intensive for water and wastewater treatment, but due to its use of low temperatures, it allows the utilization of waste heat. Lokare et al.^33^ identified the potential use of waste heat from the exhaust stream of a natural gas compressor station to apply the DCMD process for produced water treatment. Dow et al.^34^ built a pilot plant located at a gas-fired power station, which supplied waste heat to the DCMD system for wastewater treatment. Morciano et al.^35^ used small-scale desalination devices for freshwater production powered by waste heat from electric power generators. The MD process was powered by low-grade (below 80 °C) waste heat recovered from the coolant circuit of small diesel engines for electricity production. In the case of landfill leachate treatment, Yan et al.^31^ identified, through energy consumption analysis, that waste heat from incineration power plants was sufficient to drive on-site landfill leachate treatment via the DCMD process. Zico et al.^32^ used solar heating to demonstrate that this heat source was applicable for treating landfill leachate using MD. This demonstrated reductions in the operation expense (OPEX) values, thereby decreasing the total treatment costs.

The heat required for the MD process operation in landfill leachate treatment could be obtained through biogas production at the MSW landfills. As the population in urban areas grows, the volume of MSW tends to increase, as well. In Brazil, MSW is primarily composed of organic compounds,^36^ leading to higher biogas production in landfills. The energy generated from this resource is commonly applied on-site, and the quantity of waste heat generated is considerable. Although waste heat can be utilized for various applications, it is preferable to use it on-site because of the remote location of landfills relative to industries and urban centers. However, the application of biogas-derived waste heat in the MD process has not been evaluated, and it remains unclear whether the heat generated on-site is suitable for landfill leachate treatment.

Inspired by previous studies, this work investigates the utilization of waste heat recovered from engines powered by landfill biogas as a heat resource for the MD process in the landfill leachate treatment. While membrane distillation has been explored for leachate treatment and low-grade or waste heat sources have been proposed to power MD systems, the specific integration of MD with exhaust heat from landfill biogas engines has received little attention in the literature. This study aims to address this gap by evaluating the technical and energy performances of such an integrated system. The MD process was evaluated in terms of the conductivity, COD, and ammonia retention. Raw landfill leachate from a sanitary landfill in Belford Roxo, Rio de Janeiro, Brazil, was used in the experiments. A mathematical model based on the heat and mass transfer processes was developed to assess the energy consumption for landfill leachate treatment in terms of treatment capacity.

## 2 Results and Discussion

### 2.1 Experimental Tests

Operational parameters, such as the feed stream temperature and flow rate, were studied to evaluate the performance and determine the operational conditions for the DCMD process in landfill leachate treatment. Experiments with synthetic landfill leachate were conducted by varying the feed temperature from 50 to 77 °C and the feed flow rate from 60 to 300 L/h.

Figure 1 A ,B shows an increase in the permeate flux with increasing feed temperature and flow rate, respectively. However, the gains obtained by augmenting the feed flow rate were lower than those obtained by increasing the temperature, which can be explained by the exponential correlation between the temperature applied to the feed stream and vapor pressure, which is the driving force for water permeation. Alkhudhiri and Hilal^15^ reported that an increase in the temperature difference across the membrane positively affected the diffusion coefficient in the liquid phase, leading to a higher permeate flux.

**Figure 1:**
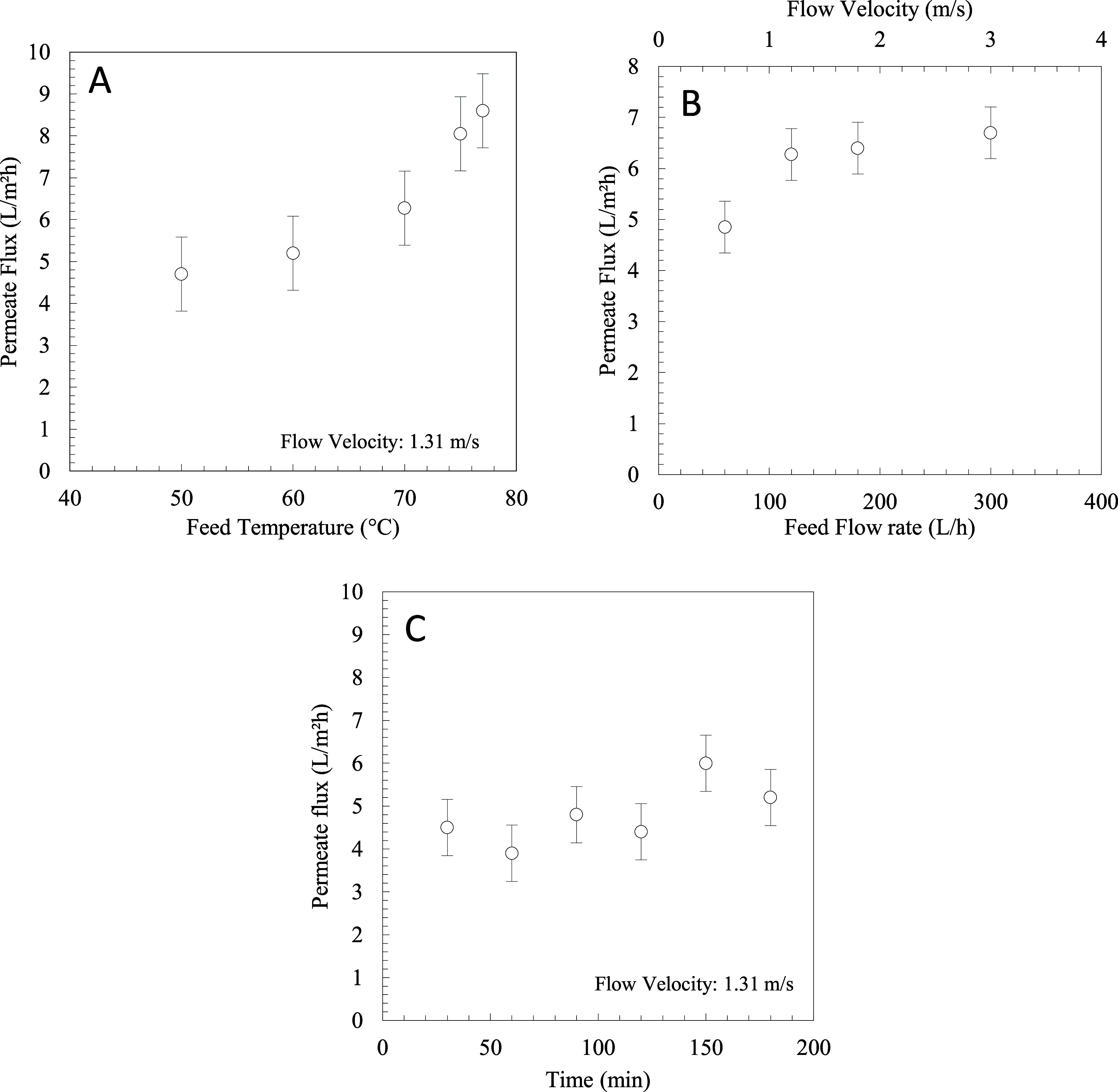
Permeate flux obtained with synthetic landfill leachate against (A) feed temperature (q_feed_: 120 L/h; q_cold_: 900 L/h), (B) feed flow rate (T_feed_: 70 °C, and ΔT_MD_: 15 °C), and (C) time (T_feed_: 70 °C, and ΔT_MD_: 15 °C, q_feed_: 120 L/h; q_cold_: 900 L/h).

The slight increase in permeate flux caused by an increase in the feed flow rate was expected because of the improved mass and heat transfer in the liquid phase, reducing the boundary layer near the membrane surface and hence the effects of temperature and concentration polarization.^15,43,44^ As one may observe, typical asymptotic rises in permeate flux were noted at flow rates above 120 L/h.^45–47^

As shown in Figure 1C, the permeate flux remained nearly constant throughout the experimental period, with an average permeate flux of 4.8 L/m^2^ h and water recovery of 72.5%, indicating that the water activity was not significantly affected by the increase in the solute concentration. Cho et al.^48^ also reported similar results by running DCMD experiments with 2 to 200 mg/L humic acid (HA) in the feed stream. Similarly, Khayet^49^ investigated DCMD with 30,000 mg/L HA and two different membranes (PTFE and PVDF) and observed a small drop in permeate flux (lower than 5%) during the experimental period. Conversely, in pressure-driven processes, such as reverse osmosis, the drop in the permeate flux may reach 90% in a short period (30 min). Even though pressure-driven separation processes may offer a higher initial permeate flux, a rapid flux drop is expected owing to the intense formation of foulant layers, indicating that DCMD is well-suited for treating this class of wastewater.

Characteristics of landfill leachate are dependent on the physical–chemical parameters, and the characterization of the synthetic and raw landfill leachate is shown in Table 1.

**Table 1:** Characterization of the Raw Landfill Leachate Permeate from the DCMD Process

parameter	synthetic feed	synthetic permeate	synthetic retention	raw feed	raw permeate	raw retention
pH	8.11 ± 0.45	8.28 ± 0.51		7.43 ± 0.48	8.67 ± 0.57	
turbidity (NTU)	13.2 ± 0.27	0.5 ± 0.54	96.60%	2.4 ± 0.38	0.7 ± 0.69	70.40%
conductivity (μS/cm)	9932 ± 26.26	512.4 ± 52.61	94.84%	10,720 ± 53.55	460 ± 59.41	95.71%
COD (mg/L)	204.0 ± 186.76	<0.5^a^		666.7 ± 33.33	76.8 ± 4.65	88.48%
ammoniacal nitrogen (mg/L)	714 ± 10.52	81 ± 12.94	88.6%	851.31 ± 12.43	198.8 ± 23.91	76.64%

a Equipment limit detection.

For synthetic and raw landfill leachates, the ionic conductivity of the accumulated permeate stream was significantly reduced with retention above 95%, indicating the effective rejection of dissolved ions present in the feed stream. Nevertheless, volatile components present in the feed stream, such as ammonia and carbonates, can permeate through the membrane, increasing the conductivity and the pH of the permeate.

The concentrations of ammoniacal nitrogen in the permeate streams of synthetic and raw landfill leachate were 81.0 and 198.8 mg/L, respectively, corresponding to retention rates of 88.6 and 76.6%. These values exceed the maximum limit of 20 mg/L for effluent discharge established by Brazilian legislation (CONAMA Resolution No. 430/2011). In the MD process, only vapor passes through the membrane. Consequently, ammonia transfer is governed by the concentration of free ammonia (NH_3_) in the feed solution, which depends on pH, temperature, and carbonate levels. Both synthetic and raw leachates exhibit alkaline pH, and the elevated feed temperature required to enhance water permeation in MD shifts the ammonia–ammonium equilibrium toward free ammonia, increasing its volatilization and transport across the membrane.

Current efforts to treat landfill leachate are limited to ammonia removal. In the MD process, the hydrophobic membrane allows volatile compounds, including free ammonia, to pass to the permeate side, necessitating a post-treatment or recovery strategy to meet the discharge limits established by Brazilian legislation. Conventional removal methods such as air stripping and nitrification–denitrification are widely used, with the latter converting ammonia into nitrogen gas (N_2_), which is released into the atmosphere.^50,51^ For ammonia recovery, traditional approaches, including struvite precipitation, are common; however, these methods suffer from high salt consumption, low recovery efficiency, and challenges in maintaining product purity.

Membrane-based technologies, particularly membrane contactors (MCs), have emerged as promising alternatives for ammonia recovery. Membrane contactor systems demonstrate high removal and recovery rates^52^ and have been extensively studied for real wastewater applications.^53–55^ The MC is advantageous when low-grade heat is unavailable, making it a more practical solution for efficient ammonia removal and recovery.^56,57^ The inclusion of an integrated ammonia removal or recovery step would increase the gross energy demand of the proposed MD-based treatment system. However, the current literature indicates that this increase does not necessarily translate into a prohibitive rise in specific energy consumption. Yang et al.^58^ demonstrated that in integrated two-stage membrane distillation systems for simultaneous water and ammonia recovery, the dominant contribution to energy demand is associated with the latent heat of water transport, whereas ammonia separation itself represents a minor fraction of the total thermal requirement. Moreover, the energetic penalty associated with ammonia recovery can be substantially mitigated through process integration and the use of low-grade or residual heat sources. Zico et al.^32^ demonstrated that when membrane-based ammonia recovery is driven by renewable or waste heat (e.g., solar or exhaust gases), the effective operational energy demand is significantly reduced, preserving system competitiveness. Therefore, although the addition of an ammonia removal or recovery step would increase the apparent specific energy consumption of the overall process, integrated thermal management and ammonia valorization strategies can keep this increase moderate.

The COD removal rate exceeded 88.45%, suggesting that the membrane successfully retained a significant portion of organic compounds. However, a small fraction of the organics detected in the permeate may be attributed to volatile compounds such as carbonates and volatile fatty acids. Volatile compounds in landfill leachates primarily arise from biogas production through anaerobic and aerobic degradation processes. The nonmethane volatile components in biogas typically exist at concentrations lower than 1% by volume. Globally, the concentration of volatile compounds in landfill leachate can range from 1 to 10^8^ ng/L.^59^

Figure 2 exhibits the DCMD permeate fluxes for raw and synthetic landfill leachate over time. Notably, the permeate flux using raw landfill leachate was slightly higher than that of the synthetic solution, but both had the same behavior over time. This difference may be associated with the difference in turbidity between synthetic and raw landfill leachates. The synthetic solution was freshly prepared, and some suspended particles could be present owing to slow dissolution kinetics, as demonstrated in Table 1. However, raw landfill leachate is collected from a stabilization lagoon in a mature landfill, and most of the suspended solids have already been settled. The presence of suspended solids may create additional transport resistance on the membrane surface, thereby reducing the permeate flux. It is important to emphasize that this surface deposition was reversible and the membrane properties were completely recovered after a single wash procedure. It should be noted that the relatively short experimental duration (180 min) is insufficient to evaluate the long-term performance under real landfill leachate operation. Aspects such as flux stability, membrane wetting, and scaling/fouling development cannot be adequately assessed within this time frame. Therefore, the reported results of simulations should be interpreted only as short-term performance indicators and do not necessarily reflect the long-term operational behavior.

**Figure 2:**
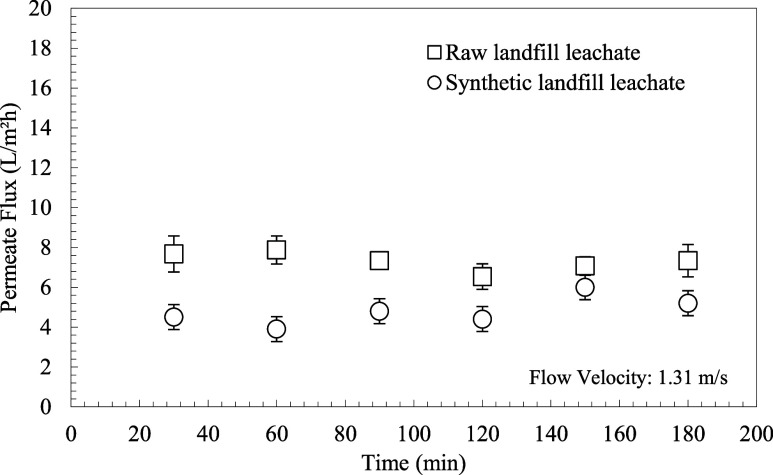
Behavior of permeate flux of raw and synthetic landfill leachate over time applying DCMD (T_feed_: 70 °C, and ΔT_MD_: 15 °C, q_feed_: 120 L/h; q_cold_: 900 L/h).

### 2.2 Simulation Tests

Figure 3 presents a comparison between the experimental DCMD data for a 2.5 wt % salt solution and the model predictions obtained from the coupled heat and mass transfer equations implemented in MATLAB. The model reproduces the expected exponential behavior of the permeate flux, showing strong agreement with the experimental data, as indicated by a low root-mean-square error (RMSE) of 0.47 relative to the data range and a high coefficient of determination (*R* ^2^ = 0.97). For three out of four evaluated temperatures, the model prediction error was found to be less than the experimental uncertainty. Although one condition exhibited an RMSE slightly higher than that of the associated experimental error, this deviation is isolated and does not compromise the overall agreement between the model predictions and the experimental data.

**Figure 3:**
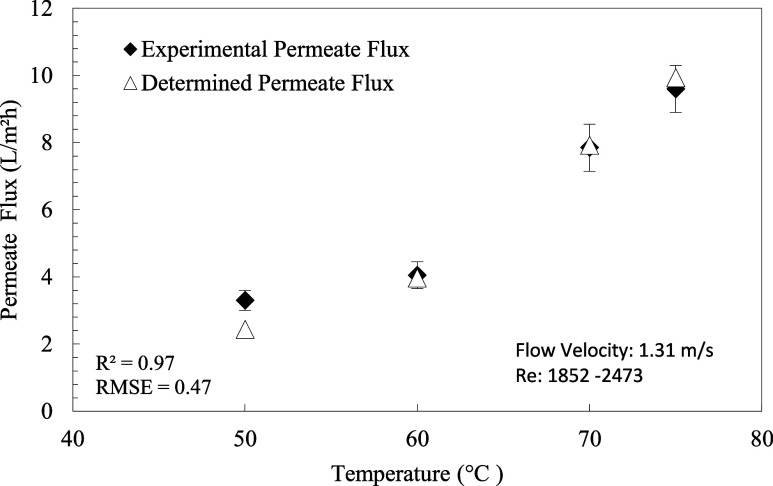
Experimental and calculated permeate flux as a function of the feed temperature (q_feed_: 120 L/h; q_cold_: 900 L/h, C_feed_: 3.5% wt. of NaCl).

The feed and permeate temperatures were determined at the membrane surface based on the algorithm shown in Figure S1, allowing calculation of the temperature polarization coefficient (TPC). Overall, for the operating temperature range, the TPC was low (0.994 at 63.6 °C), which may be attributed to the high heat transfer coefficients achieved due to the elevated feed and permeate flow rates applied in the simulation (120 and 900 L/h, respectively).^60–62^

An increase in the membrane heat transfer coefficient was also observed, which was related to an increase in the bulk feed temperature. This behavior might be expected considering the thermal conductivities of both water vapor and polymer, which are dependent on the temperature. Qtaishat et al.^63^ also considered that the higher water vapor concentration in the membrane pores due to the feed temperature increase leads to an increase in the membrane heat transfer coefficient.

The thermal efficiency of the DCMD process is shown in Figure S2 in the Supporting Information. The thermal efficiency of DCMD can be specified as the ratio of the latent heat of vaporization to the total heat (latent and conduction). The enhancement of thermal efficiency was achieved with an increase in the feed bulk temperature, which means that an increase in the evaporation rate contributes to the overall heat transfer.^61^

A DCMD process simulation was performed to evaluate the heat energy required for landfill leachate treatment. For all analyses, the feed and cold streamflow rates were considered the same, and their temperatures were fixed at 70 and 25 °C, respectively.^64^ The simulated landfill leachate was composed of 35,000 mg/L salts and 2000 mg/L organics. The landfill leachate’s physical properties were modeled using NaCl and sucrose due to their well-documented characteristics. It is important to emphasize that this representation constitutes a simplification intended to facilitate process-level analysis. Real landfill leachate exhibits significantly greater chemical complexity, including the presence of humic substances, multivalent ions, ammonia, and volatile compounds, as well as a higher fouling propensity. Although sodium humate represents the organic component of the synthetic solution, its limited physicochemical data led to the selection of a polysaccharide (sucrose) to simulate the organic portion instead. The feed tank volume was assumed to be 100 m^3^. The characteristics of the membrane used in the simulation were the same as those used in the experiments and are shown in Table S1.

The feed and permeate streams were simulated in cocurrent and countercurrent arrangements. Thus, the permeate flux was simulated as a function of feed flow rates, ranging from 20 to 60 m^3^/h, and membrane areas, ranging from 100 to 700 m^2^, as shown in Figure 4A after 200 h of operation. A decrease in the average permeate flux was observed for larger membrane areas (Figure 4A). The increase in water recovery was directly associated with an increase in the membrane area and flow rate applied during the process (Figure 4B). An asymptotic plateau for water recovery is observed at low flow rates, which is related to temperature polarization in the thermal boundary layer.^43^ This behavior was expected because, similar to what happens in a heat exchanger, the fluid flow arrangement may improve the temperature difference through the membrane and the process driving force, reflecting directly on the permeate flux.^65,66^ In Figure 4C,D, for countercurrent and cocurrent arrangements, respectively, the simulated temperature profile is shown as a function of the module length for a flow rate of 40 m^3^/h. As expected, during cocurrent operation, the maximum driving force occurred at the module inlet, followed by a progressive decrease along the membrane length (or membrane area). In contrast, during countercurrent operation, the driving force remained nearly constant along the module.^66^ Although there was a decrease in permeate flux in both flow arrangements, the average permeate flux in the countercurrent configuration was higher than that in the cocurrent flow configuration. In Figure 4A, it is noteworthy that the difference between the flow arrangements is less pronounced with increasing flow rates. This means that the operation of the DCMD with high flow rates (higher *Re* number) increases the convective heat transfer coefficient, which makes the heat transfer through the membrane the limiting step of the process, thereby reducing the influence of the fluid flow configuration.^67^

**Figure 4:**
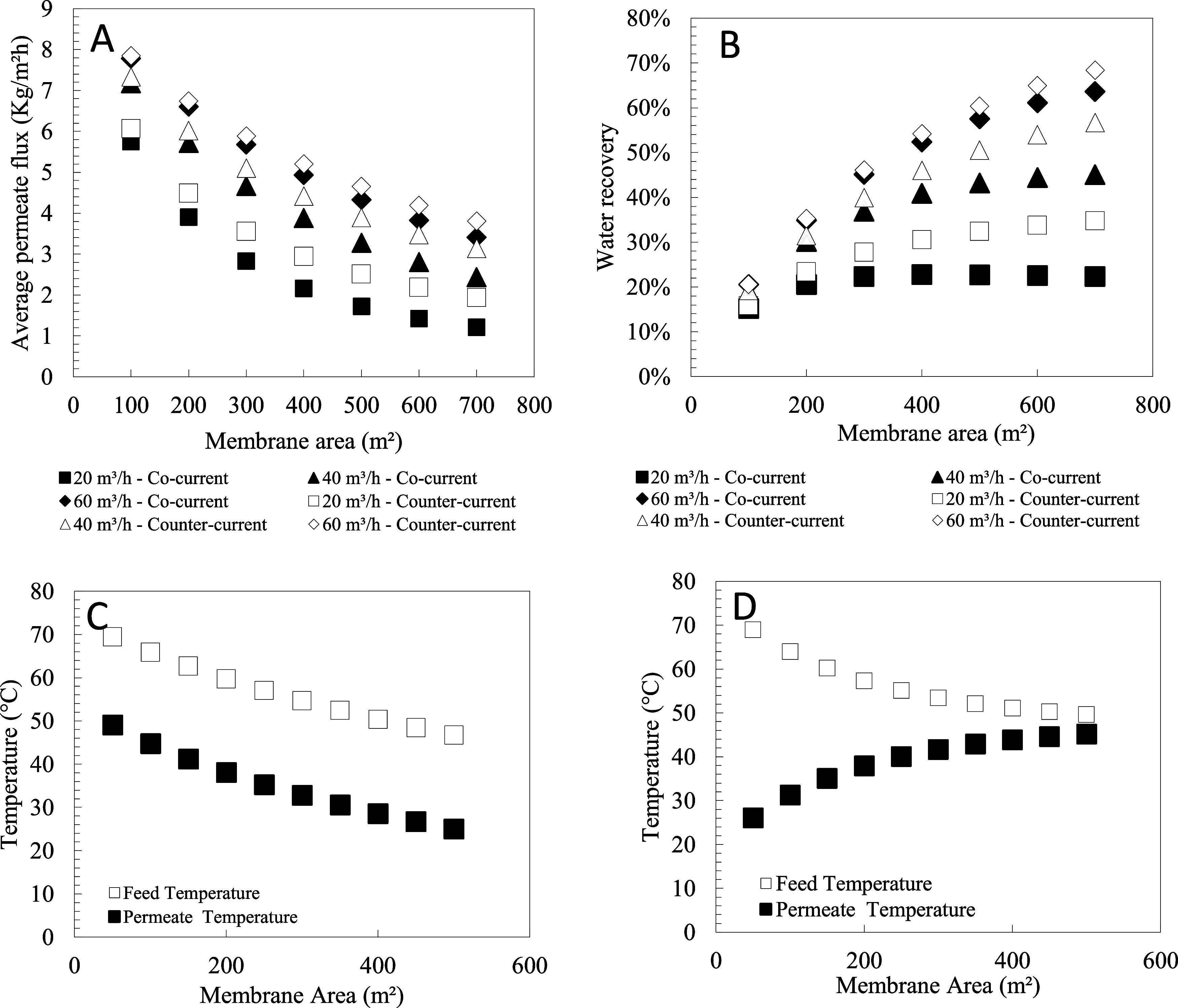
Influence of the membrane area on (A) average permeate flux (Δ*T*_MD_ = 45 °C), (B) water recovery (Δ*T*_MD_ = 45 °C), (C) temperature profile for countercurrent (q_feed_ = q_cold_ 40 m^3^/h and membrane area: 500 m^2^), and (D) cocurrent flow at 40 m^3^/h configuration current (q_feed_ = q_cold_ 40 m^3^/h and membrane area: 500 m^2^).

As demonstrated in this work, for a given amount of water recovered from landfill leachate, an increase in the flow rate leads to a reduction in the required membrane area. However, higher flow rates also demand more energy, which requires evaluation of the process performance using both SEC and GOR indicators. When the MD system operates without an energy recovery mechanism, the GOR values typically remain below 1. However, with the inclusion of energy recovery, whether through thermal isolation or feed recirculation, GOR values above 1.5 can be achieved.^31,65,68–70^

The GOR values achieved by the DCMD system for landfill leachate treatment ranged from 2.54 to 2.09 for the cocurrent flow mode and from 2.85 to 2.12 for the countercurrent flow mode. The GOR values exceeding 1 can be attributed to the implementation of partial feed recycling, which involves returning a fraction of the concentrated stream to the feed tank during the MD process. This strategy contributes to reducing the energy demand and enhancing energy recovery within the system.

The SEC values observed in both flow arrangements ranged from 0.83 to 1.12 MWh/m^3^, which aligns with findings from previous studies.^71–74^ Notably, the implementation of partial feed recycling in the simulation resulted in a 25% reduction in the energy demand. In contrast, SEC measured during experimental tests with raw and synthetic landfill leachates was approximately 2.90 and 2.84 MWh/m^3^, respectively. It is important to highlight that the simulated process configuration incorporated partial feed recycling, which significantly contributed to the GOR, whereas the experimental setup operated in a single-stage configuration and did not have any form of energy recovery during the process. Furthermore, the simulation results should be interpreted as baseline estimates as fouling and scaling phenomena were not included in the model. Under prolonged operation, inorganic precipitation and organic fouling can reduce the permeate flux and increase thermal resistance, which would likely lead to higher actual SEC values than those predicted.

Figure 5 shows a simulation of the concentration increase of the representative solutes, sucrose and NaCl, during operation for 200 h in the countercurrent configuration without considering the effects of fouling or scaling on the membrane surface. In this simulation, the feed flow rate was 40 m^3^/h and the membrane area was 500 m^2^. It was observed that a steady-state regime was achieved close to 50–60 h, where the concentrations of NaCl and sucrose almost doubled, from 35,000 and 2000 to 69,104 and 3940 mg/L, respectively. The increase in concentration on the feed side results in a reduction in water activity, which is directly related to the water vapor pressure and implies a variation in the driving force of the process.

**Figure 5:**
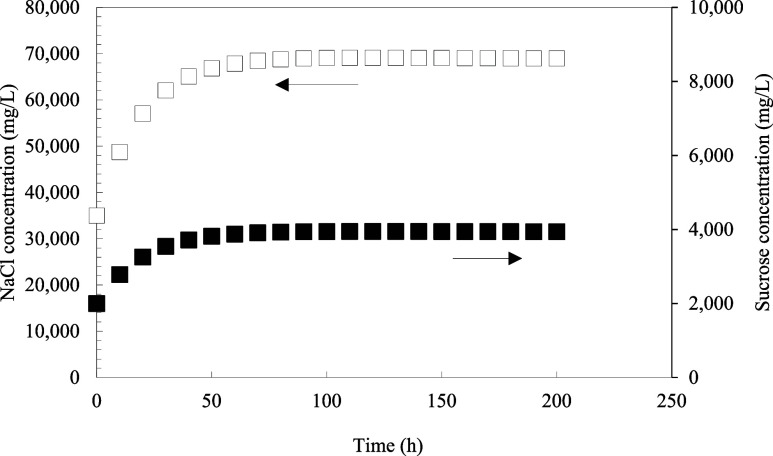
Increase in solute concentration during countercurrent operation of MD (Δ*T*_MD_ = 45 °C, q_feed_ = q_cold_ = 40 m^3^/h; membrane area: 500 m^2^).

### 2.3 Evaluation of Applying Waste Energy from Exhaust Gas in the Biogas Power Plant from Landfills for the DCMD Process

In the current literature, SEC values for MD processes show significant dispersion, depending on the membrane type, operating conditions, module design, plant scale, and the presence or absence of energy recovery systems.^75,76^ The SEC of the DCMD systems can vary from 0.14 to 10 MWh/m^3^.^77,78^ It is essential to note that the specific energy consumption of the DCMD systems used to be high because of the significant heat loss through conduction, which can be attributed to the direct contact between the stream sides and the membrane.^75^ Applying other membrane distillation configurations, such as air gap membrane distillation (AGMD) and vacuum membrane distillation (VMD), may be an alternative to improve the performance of the process without requiring supplementary energy. Yan et al.^31^ identified that the daily water recovery from landfill leachate using waste energy from an incineration treatment plant for the DCMD system was the lowest among the MD configurations compared to them (VMD and AGMD). The AGMD configuration achieved the highest performance with a daily recovery rate of approximately 92%. The high SEC values in the AGMD are attributed to the additional heat transfer resistance from the gap between the streams and the membrane. However, more membranes are required, owing to the additional mass transfer resistance.

In this work, the feasibility of using waste heat as an energy source for the DCMD process was investigated. The DCMD configuration selection can be justified under the specific operational and economic conditions. DCMD offers a simpler system design, lower capital and maintenance costs, and ease of integration with low-grade or waste heat sources, making it particularly suitable for small-scale or decentralized water treatment applications.^79–82^ Furthermore, DCMD provides high rejection rates for nonvolatile solutes and is compatible with a wide range of feedwater types. While more advanced configurations such as AGMD and VMD demonstrate superior thermal efficiency, the operational simplicity and cost-effectiveness of DCMD remain compelling advantages in contexts where the energy input is not the primary constraint.

The LandGEM methodology (eq 20) was used to simulate methane production in Brazilian landfills. The methane flow rate (Q_CH_4__) and the energy available for power generation were calculated for each site and are listed in Table 2.

**Table 2:** Scenario for the Determination of Annual Methane Generation in Brazilian Landfills

landfill	state	initial operation	service life (yrs)	yearly MSW intake (T/year)	landfill leachate generation (m^3^/day)	power generation capacity by biogas (MW)	waste energy available in exhaust gas (MW)	refs
Alfenas	Mina Gerais	2012	30	66,747	26	1.55	0.47	^89^
São Carlos	São Paulo	2012	22	73,188	59	1.70	0.51	^89^
Quatá	São Paulo	2012	20	117,849	64	2.74	0.82	^89^
Santa Maria	Rio Grande do Sul	2008	30	149,895	113	5.14	1.54	^89^
Giruiá	Rio Grande do Sul	2011	20	115,566	103	3.04	0.91	^89^
São Leopoldo	Rio Grande do Sul	2011	20	345,102	174	9.08	2.72	^89^
Guamá	Pará	2015	15	493,599	113	6.32	1.89	^89^
Battre	Bahia	1999	20	1,040,898	777	52.66	15.80	^89^
Seropédica	Rio de Janeiro	2012	20	3,600,000	1000	137.47	41.24	^64,90^
Sabará	Minas Gerais	2010	20	1,224,000	600	56.74	17.02	^50,91^
Campos	Rio de Janeiro	2009	30	154,800	200	7.52	2.26	^92,93^
Piraí	Rio de Janeiro	2000	30	7200	3	0.44	0.13	^36,92^
São Gonçalo	Rio de Janeiro	2014	20	445,680	120	15.55	4.66	^92,94^
Visconde de Rio Branco	Minas Gerais	2004	15	9000	0.5	0.34	0.10	^36^

The methane generation potential (L_0_) was assumed to be 170 m^3^ of CH_4_ per ton of organic waste based on Clean Air Act (CAA) assets (Table S5) for moderately degradable waste. The L_0_ value can range from 5 to 300 m^3^ of CH_4_ per ton of organic waste, depending on the type of MSW (relatively inert, moderately degradable, or highly degradable). The decay constant (k) was taken as 0.05 yr^–1^ due to high precipitation in this Brazilian region. The concentration of nonmethane organic compounds (NMOCs) was not available; therefore, 600 ppm hexane was used. The methane content was assumed to be 55%, and the remaining portion was carbon dioxide.

The annual methane generation was estimated on the basis of the average waste age from the start of landfill activities until closure. The lower heating value (LHV) of biogas represents the energy content released during biogas production, and it is directly linked to its methane content and inversely to its carbon dioxide content.^83^ The LHV value adopted to determine the total energy obtained from biogas was 6.5 kWh/m^3^.^84^

Electricity generation from biogas is a widely used and advantageous practice. This can be achieved by burning gas in devices such as internal combustion engines, gas turbines, or microturbines. The US Environmental Protection Agency (EPA) (EPA and Change Division 2020) states that internal combustion engines are commonly used in biogas applications because of their relatively low cost and high efficiency. These engines are often used in landfills, where biogas production can generate electric energy ranging from 3 MW to 800 MW. Multiple engines can be used for the larger projects.

Internal combustion engines typically exhibit an efficiency of 30 to 40%, meaning that only a portion of the energy supplied by fuel is converted into useful work,^85,86^ with the remaining energy dissipated primarily through the cooling system, exhaust gases, or incomplete combustion. Among these loss mechanisms, exhaust gases represent a significant source of potential energy recovery, as they carry away a considerable amount of heat from the combustion process.^87^ Without effective energy recovery systems, this thermal energy is lost. In this study, the engine capacity at each site was determined and is listed in Table 2, assuming an efficiency (η) of 30%, implying that 30% of the total energy input is converted into work, while the remaining 70% is lost, with approximately 30% of these losses carried by the exhaust gases.^84^ This exhaust heat was considered the thermal energy source for the DCMD process applied to landfill leachate treatment.

Leachate production and the power generation in landfills are intrinsically associated with the volume of solid waste received. As the amount of waste increases, so does the potential for biogas generation, thereby enhancing the landfill’s power generation capacity. Simultaneously, higher waste inputs lead to increased leachate generation due to intensified organic decomposition and elevated moisture content.^36^ This behavior is illustrated in Figure 6, which presents data from selected landfills from Brazil. The figure reveals a correlation between landfill leachate production, the municipal solid waste intake, and the recoverable energy from exhaust gases after electric power generation using biogas resource.

**Figure 6:**
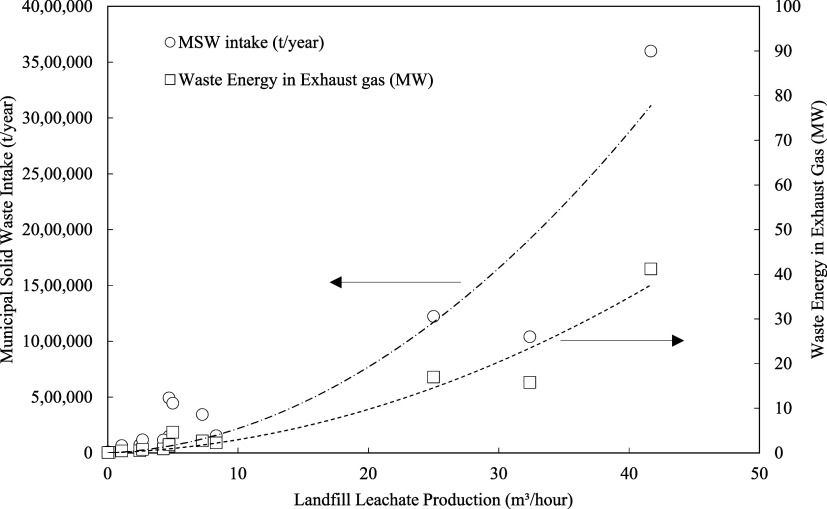
Relationship between municipal solid waste intake, waste energy potential, and landfill leachate production in Brazilian landfills.

The analysis of SEC presented in Figure 7 is based on DCMD simulation results discussed in Section 3.2, along with a comparison to the available specific energy derived from waste heat utilization and full biogas generation, as shown in Figure 6. The chart suggests that complete landfill leachate treatment could be achieved when exhaust gas is employed as the heat source for the MD process.

**Figure 7:**
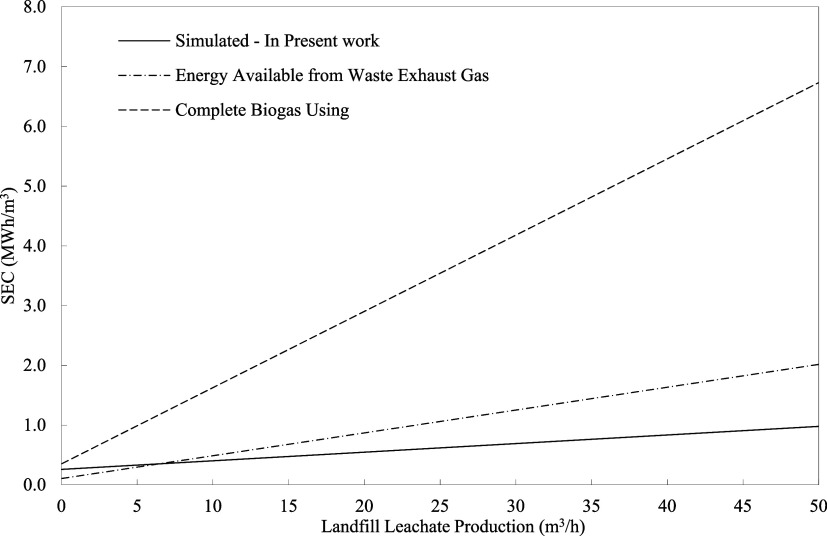
Specific energy consumption in selected Brazilian landfills using DCMD and specific energy available using waste heat or full biogas utilization.

The available SEC values represent the upper limit of energy consumption that the selected landfills may incur for leachate treatment using both waste heat from exhaust gases and energy from biogas generation. The simulation-derived SEC values served as reference parameters for the analysis of the landfill performance curves. This implies that the simulation outcomes are representative of the landfills examined in this study under the assumption of a DCMD configuration with partial feed recycling.

High SEC values indicate that a significant amount of waste energy is available at the site for treating landfill leachate via the membrane distillation process. For instance, the Battre, Seropédica, and Sabará landfills have 1.34, 1.70, and 1.06 MW of waste energy available, respectively, per cubic meter of leachate treated, which are far above the DCMD energy requirement. Conversely, landfills with leachate production rates below 6.4 m^3^/h require supplementary energy sources or partial allocation of energy from biogas-based power generation to achieve complete landfill leachate treatment using a DCMD system with partial feed recycling. This suggests that the DCMD process with energy recovery is more effective in larger landfills, where higher municipal solid waste intake enhances biogas production and, consequently, waste heat availability.

In a scenario where the entire biogas output is dedicated exclusively to landfill leachate treatment—rather than diverted for power generation—all evaluated sites can achieve complete treatment through membrane distillation without incurring external energy costs. Although biogas has alternative uses, such as electricity generation or upgrading to biomethane for sale, the latter requires additional logistics and purification steps that increase operational costs.^88^ For smaller landfills, prioritizing thermal energy recovery is generally more advantageous than electricity generation, due to the relatively low power output and high grid connection costs, which depend on factors such as proximity to the gas grid and local energy markets.

## 3 Conclusions

The viability and performance of DCMD for landfill leachate treatment in sanitary landfills were investigated. Operational parameters, including the feed stream temperature and flow rate, were evaluated. The increase in the feed stream temperature and flow rate positively affected water production during the process. The presence of volatile components was identified by ammoniacal nitrogen, indicating that pretreatment or post-treatment is necessary for the application of the MD process.

The model indicated that lower transmembrane temperature differences corresponded to higher temperature polarization coefficients. Conversely, higher transmembrane temperature differences reduce temperature polarization effects and improve the thermal efficiency of the process.

The integration of MD into sanitary landfills shows that the performance of the countercurrent DCMD is better than that of the cocurrent. Nevertheless, the influence of the flow arrangement is not significant for high flow rates. The simulation used partial feed recycling, which resulted in an energy gain for the process and improved the GOR to 2.85, reducing the specific energy consumption by 25%.

The potential of utilizing waste heat from internal combustion engines in landfills to drive the MD process for the treatment of landfill leachate was explored. With engine efficiencies around 30%, approximately 70% of the input energy is lost, of which about 30% is carried away by exhaust gases, representing a significant opportunity for energy recovery. A correlation was observed among landfill size, biogas generation, and leachate production, with larger landfills offering greater potential for energy recovery and wastewater treatment. Simulation results indicate that landfills producing more than 6.4 m^3^/h leachate can meet treatment demands using only on-site waste energy, while smaller sites may require supplemental energy. Notably, if all biogas is allocated, a complete leachate treatment is achievable at all studied sites without external energy input, highlighting the feasibility of membrane distillation in landfill operations.

## 4 Methodology

### 4.1 Materials

A commercial direct-contact module was used owing to its simplicity in operating and controlling the process parameters. The module, manufactured by Microdyn (MD020CP-2N), contains 40 hydrophobic polypropylene (PP) hollow fibers and a total interfacial area of 0.1 m^2^. The hollow fibers have a wall thickness of 500 μm, a nominal pore diameter of 0.2 μm, and a porosity of 70%. Additional characteristics about membrane are provided in Table S1 in the Supporting Information.

### 4.2 Synthetic Landfill Leachate

Experimental tests were carried out using a NaCl (Merck) solution with a concentration of 2.5 wt % for the initial MD evaluation. The synthetic landfill leachate was prepared to contain humic acids (sodium humate), salts (NaHCO_3_), ammonium (NH_4_Cl), and a trace of minerals and metals to simulate a typical raw leachate from the landfill.^37^ The composition of the synthetic landfill leachate is shown in Table S2.

### 4.3 Raw Landfill Leachate

Raw landfill leachate was obtained from a sanitary landfill located in Belford Roxo, Rio de Janeiro, Brazil. Samples were collected and stored in plastic containers at room temperature.

### 4.4 Experimental Apparatus

The experimental apparatus, Figure 8, consists of a DCMD module, which operates in a single-stage configuration without any energy recovery, connected to two tanks with a capacity of 2 L, representing the permeate and feed sides. Each side had a centrifugal pump, a flowmeter, and a thermometer at the inlet and outlet of the DCMD module. Additionally, the permeate side was equipped with a digital balance to continuously measure permeate mass and calculate water flux based on the permeate flow rate per membrane area, as well as an ionic conductivity meter to assess permeate water quality.

**Figure 8:**
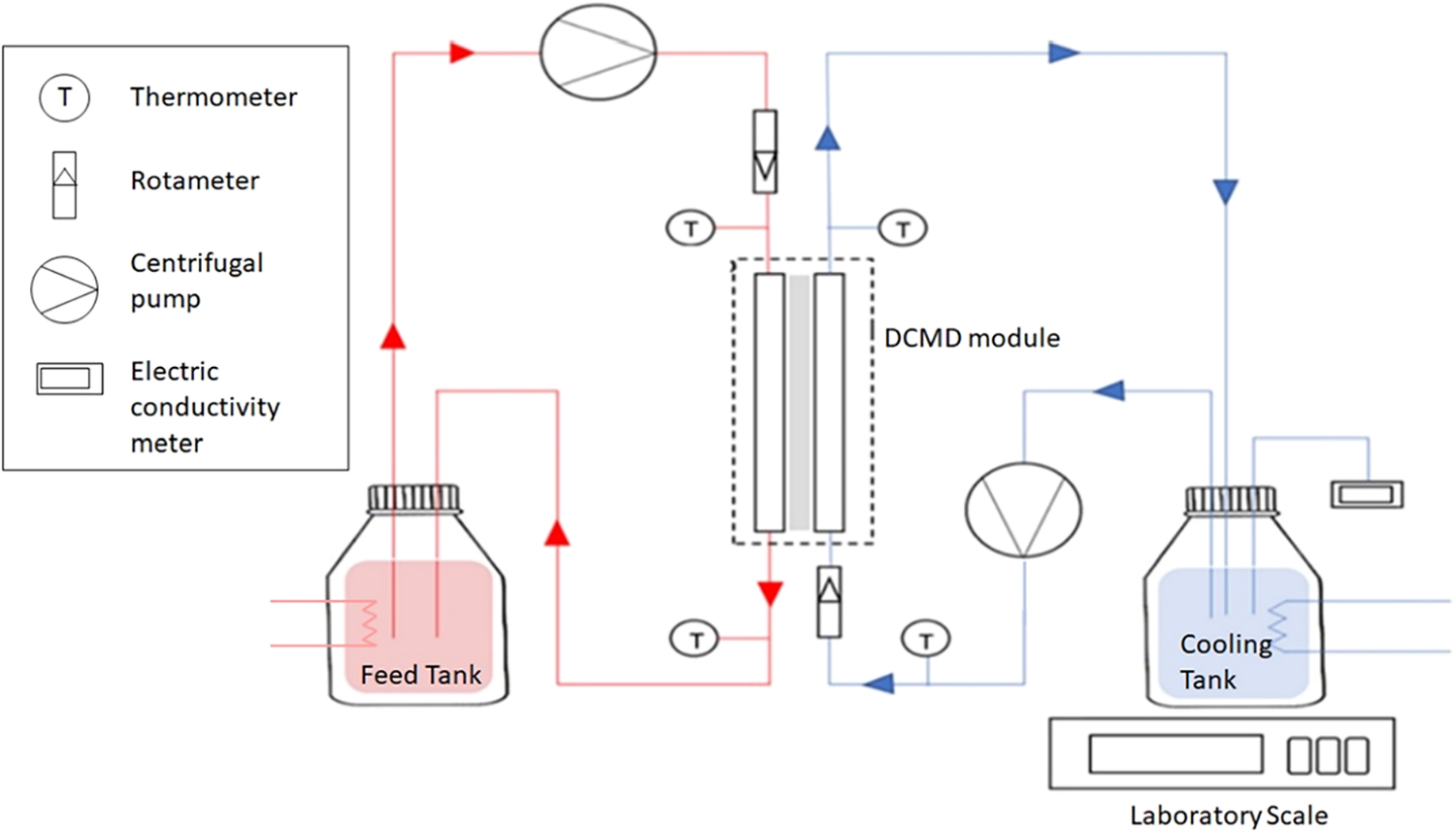
Schematic diagram of the DCMD experimental setup.

The feed solution and distilled water were heated and cooled using thermostatic baths (Cole-Parmer, 12108-00) until the bulk temperatures ranged from 50 to 77 °C on the feed and 25 °C on the permeate side. Subsequently, the solutions were pumped in countercurrent through the DCMD module. Experiments using this setup were conducted for 2 h and performed in triplicate to evaluate the experimental uncertainty.

To evaluate the removal efficiency and feasibility, samples from the feed at the beginning of each experiment and from the accumulated permeate obtained by using raw or synthetic landfill leachate were collected and characterized. The following physicochemical analyses were performed: ionic conductivity, pH, COD, turbidity, and ammoniacal nitrogen were measured according to Standard Methods for Examination of Water and Wastewater.^38^ Dilution of the permeate by distilled water initially present in the permeate tank was taken into account during the data analysis.

At the end of each test, the membrane distillation system was cleaned using distilled water to rinse the module and setup. Membrane drying was performed by passing compressed air through both the lumen and shell sides of the membrane. Subsequently, distilled water was circulated throughout the entire system for 20 min, and ionic conductivity was monitored every 5 min. The cleaning procedure was considered complete when the ionic conductivity measured at the module inlet matched that at the outlet.

### 4.5 Modeling

#### 4.5.1 Mass and Heat Transfer through the Membrane Liquid–Vapor Interface

In general, simultaneous heat and mass transfer phenomena take place through the membrane pores in all MD configurations. The permeate flux J across the membrane is proportional to the transmembrane driving force (vapor pressure) and is described by eq 1

1 $$J=C_{m}\left(P_{f}-P_{p}\right)$$

where *C*_m_ is the membrane mass transfer coefficient and *P*_f_ and *P*_p_ are the vapor pressure on feed and permeate sides of the membrane, respectively. The pressure difference represents the driving force for mass transport.

The mass transfer through the membrane is governed by the relationship between the pore size and permeating molecule diameter. The Knudsen number is defined as the ratio of the molecular mean free path (λ) to mean pore diameter (*d*_p_), eq 2, and it serves as a criterion to determine the predominant mass transport mechanism inside the membrane pore, which is assumed to be cylindrical.

2 $$\mathrm{Kn}=\frac{\lambda }{d_{p}}$$

The mean free path is given by eq 3

3 $$\lambda =\frac{K_{b}T}{\sqrt{2}{\pi \mathrm{Pd}}_{e}^{2}}$$

where *K*_b_ is the Boltzmann constant, *T* is the absolute temperature, *P* is the mean pressure inside the membrane pores, and d_e_ is the molecule diameter.

The Knudsen diffusion regime is established when the collision between molecules and membrane pore walls is favored, which occurs when the mean pore diameter is smaller than the mean free path (*Kn* ≥ 1). In contrast, when the pore diameter exceeds the mean free path (*Kn* ≪1), mass transfer is described by molecular diffusion. In the intermediate regime, both Knudsen and molecular diffusion contribute to mass transport. Accordingly, the combination of both mechanisms, as shown in eq 4, was used to determine the membrane mass transfer coefficient

4 $$C_{m}=\frac{\pi }{\mathrm{RT}\tau \delta }{\left[{\left(\frac{2}{3}\sqrt{\frac{8\mathrm{RT}}{\pi M_{w}}r_{t}^{3}}\right)}^{-1}+{\left(\frac{PD_{w}}{p_{air}}r_{t}^{2}\right)}^{-1}\right]}^{-1}$$

where *R* is the universal gas constant, *T* is the absolute temperature, τ is the tortuosity, δ is the membrane thickness, *M*_w_ is the molecular weight of water, *r*_t_ is the pore radius in the transition regime, *P* is the total pressure inside the pore, *D*_w_ is the molecular diffusion coefficient of water vapor, and the *p*_air_ is the air pressure within the membrane pore.

The heat transfer occurs in three stages: from the bulk feed to the membrane surface by convection, through the membrane by conduction and latent heat transfer associated with vaporization, and the membrane surface to the bulk of permeate by convection. Considering the heat transfer in steady-state, assuming one-dimensional, perpendicular to flow direction, and constant thermophysical properties, neglecting heat generation and loss, the heat flux (*Q*_m_) in the water channels and the membrane is described by eq 5

5 $$Q_{m}=h_{f}\left(T_{\mathrm{fb}}-T_{\mathrm{fm}}\right)=\frac{\kappa_{m}}{\delta }\left(T_{\mathrm{fm}}-T_{\mathrm{pm}}\right)+J\Delta H_{v}=h_{p}\left(T_{\mathrm{pm}}-T_{\mathrm{pb}}\right)$$

where *h*_f_ and *h*_p_ are the convection heat coefficients of the feed solution and permeate stream, respectively, expressed in W/m^2^ °C, and are determined by using eq 6. The temperatures *T*_fb_, *T*_fm_, *T*_pm_, and *T*_pb_ are the temperatures of the feed bulk, feed membrane surface, permeate membrane surface, and permeate bulk, respectively. The κ_m_ is the combined membrane thermal conductivity in W/m °C, δ is the membrane thickness in meters, *J* is the permeate flux in kg/m^2^h, and Δ*H*_v_ is the vaporization enthalpy in kJ/kg.

6 $$h_{f,p}=\frac{Nu_{f,p}\kappa_{f,p}}{d}$$

*Nu* and κ are the Nusselt number and thermal conductivity of the feed solution and permeate stream, respectively, and *d* is the characteristic size of the channels in meters. There are many ways to determine Nu according to the type of flow regime (laminar or turbulent).^39^ In this work, the *Nu* used is given by eq 7 and is valid only for a Reynolds number (*Re*) range from 200 to 3000.^40^

7 $$Nu_{f,p}=0.43Re^{0.591}{\mathrm{Pr}}^{0.303}$$

The conductive heat transfer coefficient of the membrane (*h*_m_) was determined using eq 8, using the combined air/polymer matrix thermal conductivity (κ_m_) and membrane thickness (δ). More details regarding the determination of combined air/polymer matrix thermal conductivity are provided in Supporting Information.

8 $$h_{m}=\frac{\kappa_{m}}{\delta }$$

The membrane liquid–vapor interface temperatures were determined by relating the permeate flux to the bulk temperatures through eqs 9 and 10.

9 $$T_{\mathrm{fm}}=\frac{T_{\mathrm{fb}}h_{f}+h_{m}\left(T_{\mathrm{pb}}+T_{\mathrm{fb}}\frac{h_{f}}{h_{p}}\right)-J\Delta H_{v}}{h_{f}\left(1+\frac{h_{m}}{h_{p}}\right)+h_{m}}$$

10 $$T_{\mathrm{pm}}=\frac{T_{\mathrm{pb}}h_{f}+h_{m}\left(T_{\mathrm{fb}}+T_{\mathrm{pb}}\frac{h_{p}}{h_{f}}\right)+J\Delta H_{v}}{h_{p}\left(1+\frac{h_{m}}{h_{f}}\right)+h_{m}}$$

The membrane surface temperatures and water permeate flux were obtained using a numerical method, and the fluid properties (Tables S3 and S4) were updated for each iteration. The algorithm to determine the liquid–vapor membrane temperature and permeate flux can be seen in Figure S1 in the Supporting Information.

#### 4.5.2 Heat and Mass Transfer in the Membrane Module Flow Direction

In the feed and permeate flow directions, the membrane module was divided into n segments, each with an identical membrane permeation area, defined as dA (m^2^). Each dA has direct contact with both feed and permeate mass flow, defined by *m*_f_^·^ and *m*_P_^·^ (kg/s), with the corresponding surface temperatures *T*_fm_ and *T*_pm_, respectively.

The energy balance of the feed stream was determined by assuming that the input heat energy is equal to the difference between the energy output from the previous dA element and the heat energy transferred by conduction through the membrane. Because the conductivity heat transfer term is much smaller than the latent heat associated with vaporization,^41^ it was neglected in the calculation of outlet bulk temperature, as described using eq 11. The logarithmic average temperature between the bulk and the membrane surface temperature ( $\overset{®}{T_{f\left(n\right)}}$ ), given by eq 12, was used to determine the effective temperature along the module. The specific heat of the feed stream is indicated by *Cp*_f_ and given in J kg^–1^ K^–1^.

11 $${ṁ}_{f\left(n\right)}Cp_{f\left(n\right)}\overset{®}{T_{f\left(n\right)}}={ṁ}_{f\left(n-1\right)}Cp_{f\left(n-1\right)}\overset{®}{T_{f\left(n-1\right)}}-J\Delta H_{v}\mathrm{dA}$$

12 $$\overset{®}{T_{f\left(n\right)}}=\frac{T_{\mathrm{fb}\left(n\right)}-T_{\mathrm{fm}\left(n\right)}}{\ln\frac{T_{\mathrm{fb}\left(n\right)}}{T_{\mathrm{fm}\left(n\right)}}}$$

Because the steady-state assumption was adopted, the heat energy variation in dA at the feed side was equivalent to that at the cold stream side. This assumption was applied to determine the average temperature between the bulk and the membrane surface at the cold stream side, as indicated by eq 13. The specific heat of the cold stream is indicated by *Cp*_p_ and given in J kg^–1^ K^–1^.

13 $$\overset{®}{T_{p\left(n\right)}}=\overset{®}{T_{p\left(n-1\right)}}+\frac{{ṁ}_{f\left(n\right)}Cp_{f\left(n\right)}\left(\overset{®}{T_{f\left(n-1\right)}}-\overset{®}{T_{f\left(n\right)}}\right)}{{ṁ}_{p\left(n\right)}Cp_{p\left(n\right)}}$$

A similar assumption was applied to determine the amount of water mass transferred from the feed to the cold side through the membrane element (dA).

The variation in feed solution concentration for each membrane element (dA) was determined using eq 14, and complete salt rejection by the membrane was assumed in the modeling.

14 $$C_{\mathrm{mix}\left(n\right)}=\frac{\left(C_{\mathrm{mix}\left(n-1\right)}.{ṁ}_{f\left(n\right)}\right)}{{ṁ}_{f\left(n\right)}-J\mathrm{dA}}$$

#### 4.5.3 Membrane Distillation Integrated Process

Landfill site simulations were performed to evaluate the performance and heat energy required for application of the MD process to landfill leachate treatment, considering the following assumptions:

- Volatile components present in landfill leachate (ammonium, organic acids, etc.) were not considered in the MD simulations. These nonmethane volatile components, typically present in biogas at concentrations below 1% by volume, are not expected to have a significant impact on the overall energy demands of the membrane distillation process. Therefore, their exclusion from the simulations does not notably alter the system’s energy efficiency or performance.
- Simulation results are presented as baseline estimates as fouling and scaling phenomena were not included in the model.
- NaCl was chosen to represent the group of salts due to its well-documented physicochemical characteristics in the literature. For the organic fraction, sucrose (C_12_H_22_O_11_) was selected instead of sodium humate because of the latter’s complex nature and limited available data on its physicochemical properties. This approach intends to facilitate process-level analysis, and it is important to stress that it does not capture the compositional complexity of real landfill leachate, which contains humic substances, multivalent ions, ammonia, and other compounds that may enhance fouling and scaling.
- The fraction of concentrated leachate was returned to the equalization lagoon to control the MD feed concentration.
- Raw landfill leachate was discharged in a feed tank at constant flow rate, temperature, and concentration.
- The permeate was continuously discharged at a constant temperature.

Figure 9 represents the overall process flow. Landfill leachate reaches the feed tank (lagoon) at a determined flow rate (*q*_leach_in_), concentration (*C*_leach_in_), and temperature (*T*_leach_in_). The landfill leachate mixed with a fraction of the concentrated MD stream leaves the feed tank at a flow rate (*q*_lech_out_) in the direction of the heat exchanger to be heated to the inlet MD temperature *T*_leach_m_in_. Within the MD module, the feed stream is treated and left the module with a temperature (*T*_leach_m_out_), concentration (*C*_leach_m_out_), and flow rate (*q*_leach_m_out_) calculated using the equations described in Section 2.5.2.

**Figure 9:**
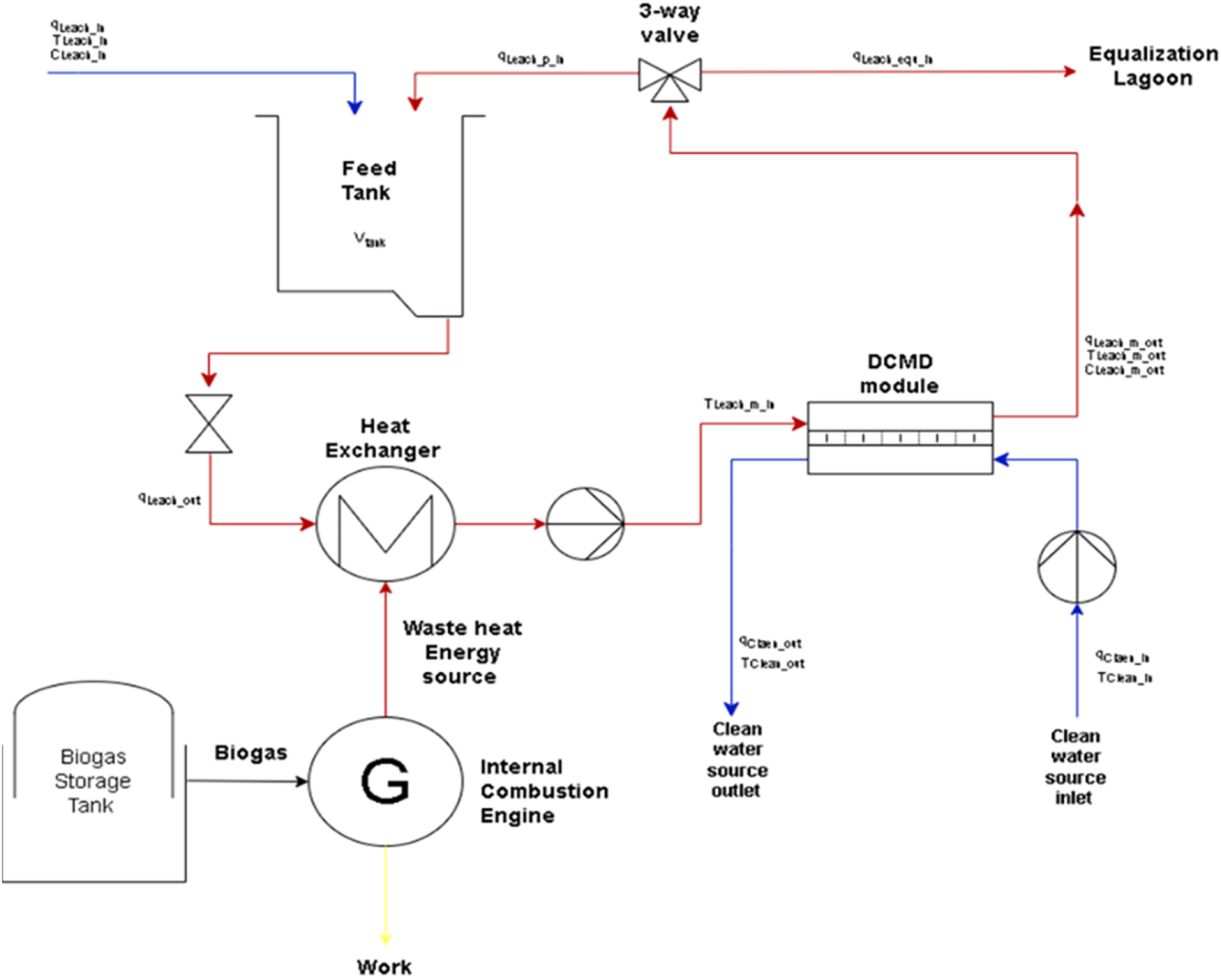
Schematic diagram illustrating the process of membrane distillation treatment designed for a landfill site.

The concentrated MD stream was pumped to a three-way valve to maintain a constant tank volume. A portion of concentrated landfill leachate was returned to the tank, while the remaining fraction was discharged from the system to the equalization lagoon.

Mass and energy balances were applied to determine the tank concentration and temperature using eqs 15 and 16.

15 $$V_{\mathrm{tank}}\frac{\partial C}{\partial t}={C_{\mathrm{leach}}}_{\mathrm{in}}{{ṁ}_{\mathrm{leach}}}_{\mathrm{in}}+{{C_{\mathrm{leach}}}_{m}}_{\mathrm{out}}{{{ṁ}_{\mathrm{leach}}}_{m}}_{\mathrm{out}}-C_{\mathrm{leac}h_{\mathrm{out}}}{{ṁ}_{\mathrm{leach}}}_{\mathrm{out}}$$

16 $${ṁ}_{\mathrm{tank}}Cp_{\mathrm{tank}}T_{\mathrm{tank}}={ṁ}_{\mathrm{leac}h_{\mathrm{in}}}C{p_{\mathrm{leach}}}_{\mathrm{in}}{T_{\mathrm{leach}}}_{\mathrm{in}}+{{{ṁ}_{\mathrm{leach}}}_{m}}_{\mathrm{out}}C{{p_{\mathrm{leach}}}_{m}}_{\mathrm{out}}{{T_{\mathrm{leach}}}_{m}}_{\mathrm{out}}$$

Water recovery (Rec_process_) is expressed by the amount of water extracted from landfill leachate by DCMD, which is determined by eq 17.

17 Recprocess=ṁpṁleachin

The process recovery is expressed as a percentage, and *m*_p_^·^ is the total mass flow rate of the permeate (kg/s) obtained through the product between the permeate flux (kg/m^2^s) and membrane area (m^2^).

The efficiency of the MD process was assessed using key parameters such as the GOR and SEC, as defined in eqs 18 and 19, respectively.

18 $$\mathrm{GOR}=\frac{{ṁ}_{\mathrm{perm}}\Delta H_{v}}{{\mathrm{Q̇}}_{\mathrm{perm}}}$$

19 $$\sec=\frac{{\mathrm{Q̇}}_{m}\rho_{w}}{\mathrm{JA}}$$

where *m*_p_^·^ is the permeate mass flow rate, Δ*H*_v_ is the specific enthalpy of evaporation, and *Q̇*_perm_ is the power input in the heater. Additionally, *Q̇*_m_ represents the total heat flux through the membrane, ρ_w_ is the water density, *J* is the permeate flux, and *A* is the membrane area.

The LandGEM first-order degradation model, described by eq 20, was utilized to estimate the generation of landfill gas and methane from MSW landfill. The methodology is based on a first-order degradation model, encompassing model parameters such as methane production potential and the first-order decay rate constant^42^

20 $$Q_{CH_{4}}=kL_{0}\sum_{i=1}^{y}\sum_{j=0.1}^{1}(\frac{M_{i}}{10})e^{-kt_{i,j}}$$

where *Q*_CH_4__ is the computed CH_4_ gas generation for a specific year *y* (m^3^ CH_4_ yr^–1^); *k* is the first-order decay rate constant (yr^–1^), *L*_0_ is the methane generation potential (m^3^ CH_4_ wet Mg^–1^), *M_i_* is the mass of waste landfilled in the *i*th year (Mg), *i* is the range from 1 to *y*, *j* is the intra-annual time increment used to calculate CH_4_ generation, and *t* is the time in years. Further details regarding these parameters can be found in the Supporting Information.

## Supplementary Material



## Notes

Microdyn MD020CP-2N membrane characteristics and synthetic landfill leachate composition; mass transfer through the membrane liquid–vapor interfaces; conductive heat transfer coefficient of the membrane equations; fluid properties; algorithm for simulation of membrane distillation applying Nusselt equations; effect of feed temperature on thermal efficiency of direct-contact membrane distillation; efficiency parameters of the membrane distillation process; landfill gas generation (LFG); and on-site waste heat recovery (PDF)
