# Supplementary material for: Technical Perspective of Landfill Leachate Treatment via Membrane Distillation Using Waste Heat from Biogas Combustion for Power Generation
Source: ACS Omega. 2026 Jul 10;11(28):41652–66. doi: 10.1021/acsomega.6c01204 (PMC13393372; doi:10.1021/acsomega.6c01204)
Supplement: Supplementary file 1 [file ao6c01204_si_001.pdf]

Supplementary material

Technical perspective of landfill leachate treatment via membrane distillation using waste heat from biogas combustion for power generation

Bruno Valim Marques da Silva[1*], Fabiana Valéria da Fonseca[1], Cristiano Piacsek Borges[2]

[1]School of Chemistry, Chemical Engineering Department, Federal University of Rio de Janeiro, Brazil

[2]COPPE/Chemical Engineering Program, Federal University of Rio de Janeiro, Brazil

Includes:

14 pages, 2 figures, and 5 tables.

[*] Corresponding author. E-mail: *bsilva@peq.coppe.ufrj.br (Bruno Valim Marques da Silva).*

Table S1 – Mycrodyn (MD020CP-2N) module characteristics

Parameter	Value
Module Length	470 mm
Membrane thickness	500 μm
Nominal pore diameter	0.20 μm
Number of fibers	40
Porosity	70%
Shell internal diameter	30 mm
Thermal conductivity of PP	0.115 $Wm^{-1}K^{-1}$
Tube outer radius (hollow fiber)	1.4 mm
Tube inner radius (hollow fiber)	0.9 mm

Table S2 – Synthetic Landfill Leachate Composition. Source:[1]

Component	Concentration [mg/L]
Sodium Humate	250
NH_4Cl	191
$CaCl_2.2H_2O$	36.7
$MgCl_2.2H_2O$	83.6
$CdCl_2.2.5H_2O$	2
$NiCl_2.6H_2O$	4
$ZnSO_4.7H_2O$	4.4
$MnCl_2.4H_2O$	3.6
$NaHCO_3$	2550

The Conductive heat transfer coefficient of the membrane

The combined membrane thermal conductivity was determined using Equation S1 and the flux law model [2], [3], considering the thermal conductivities of the polymer (κ_s) and air (κ_{air}), porosity (ε), and an intermediate factor (β) that can be calculated using Equation S2.

$$\kappa_m = \frac{\kappa_{air[1+2\beta(1-\varepsilon)]}}{1-\beta(1-\varepsilon)} \quad \text{(S1)}$$

$$\beta = \frac{\kappa_s - \kappa_{air}}{\kappa_s - 2\kappa_{air}} \quad \text{(S2)}$$

The polymer and air thermal conductivities were estimated as a function of mean membrane temperature (T_m) using Equations S3 and S4 and were valid only for a temperature range of 20–75°C [3], [4]. The membrane temperature is assumed to be the average temperature of the feed and permeate membrane surfaces.

$$\kappa_s = 0.00125T_m - 0.2351 \quad \text{(S3)}$$

$$\kappa_{air} = 0.0015\sqrt{T_m} \quad \text{(S4)}$$

Fluid Properties

The composition of landfill leachate varies from one country to another depending on the living population's standard. Additionally, time is another factor that contributes to its composition (Manjunatha et al., 2020).

Determining the physical properties of the landfill leachate is a challenge due to its heterogeneity caused by the factors described in this section.

For the purpose of applying the synthetic solution described in Table S2 on the MD process simulation, some changes to the solution characteristics were taken into consideration. From the group of salts on the solution, one salt was chosen to represent them. The NaCl was chosen due to a large amount of information about its characteristics found in the literature. The sodium humate describes the organic portion of the synthetic solution. However, due to the complexity

of the sodium humate and the absence of information about its properties, mainly physiochemical characteristics, one polysaccharide was chosen for this portion of the solution. The sucrose ($C_{12}H_{22}O_{11}$) was chosen to describe the organic portion for the same reasons of NaCl.

It did not consider the presence of volatile components such as acetic acid and ammonium on the solution because the vapor and volatile components in the feed solution cross the porous membrane in the membrane distillation process. The physical properties used to the model the NaCl solution and water can be seen in Table S3.

Table S3 – Physic-chemical Properties of NaCl solution and Water. Source: [6], [7]

**Salt Solution Properties**	**Equation**[a]
Density[b] [Kg/m³]	$\rho_{Nacl} = 980 + 1950x_{NaCl}$
Specific Heat [J/KgK]	$C_{p_{Nacl}} = 4180 - 8370x_{NaCl}$
Thermal conductivity [W/mK]	$\kappa_{Nacl} = (0.608 + 0.000764T)(1 - 0.98x_{Nacl})$
Viscosity [Pas]	$\mu_{Nacl} = (0.00087 - 0.00000063T)(1 - 12.9x_{Nacl})$
**Solution Properties**	
Density [Kg/m³]	$\rho_w = -0.0027T^2 - 0.1446T + 1001.1$
Specific Heat [J/KgK]	$C_{p_w} = 5.10^{-7}T^4 + 0.0002T^3 + 0.0386T^2 + 2.4906T + 4223.5$
Thermal conductivity [W/mK]	$\kappa_w = 10^{-8}T^3 - 10^{-5}T^2 + 0.0022T + 0.5583$
Viscosity [Pas]	$\mu_w = 3.10^{-6}T^4 - 0.0021T^3 + 0.4659T^2 - 43481T + 1763.4$

The measurement of density of sucrose solution was performed applying the Equation S5, where ρ_w is the water density, c is the concentration of sucrose (g sucrose % g of solution) and T is the temperature in Celsius degree (°C). The sucrose density equation is valid for a temperature range from 10 to 80°C and mass fraction from 5 to 85% [8].

$$\rho_{Sucrose} = \rho_w + a_1c^2 + a_3c^3 + (b_1c + b_2c^2 + b_3c^3)(T - 20) + (c_1c + c_2c^2 + c_3c^3)(T - 20)^2 + (d_1c + d_2c^2)(T - 20)^3 + e_1c(T \quad \text{(S5)}$$

The coefficients a,b,c, d and e can be seen in Table S4

Table S4: Coefficients for sucrose solution density ($\rho_{Sucrose}$)

**Coefficient**	**Value**
a1	385.85074
a2	-13.03435
a3	-3.663
b1	-0.459244
b2	0.075699
b3	0.062667
c1	0.0060198
c2	-0.0013008
c3	-0.0004907
d1	-0.00005110
d2	0.00001580

e1	0.0000001986

The specific heat and the thermal conductivity of the sucrose solution ($Cp_{sucrose}$ and $\kappa_{sucrose}$) can be expressed by Equations S6 and S7 [9]. Where the T is the temperature in Celsius degree, c is the sucrose concentration (g sucrose % g of solution). The validation of these equations is only for a temperature range from 0 to 100°C and a concentration from 0 to 90%.

$$C_{psucrose} = (4182.86 - 45.70327c) + (0.1145707 + 0.075577c)T \quad \text{(S6)}$$

$$\kappa_{sucrose} = (0.2145613296 + 0.001311369T) - (0.001479787 + 6.1488.10^{-6}T)c \quad \text{(S7)}$$

The viscosity of sucrose solution was expressed in Equation S8. This equation is applied to the calculation of the viscosity of sugar solutions in the range of concentration from 0 to 85% (w/w) and the temperatures between 10 and 80°C [10].

$$loglog\ \mu = 22.46N - 0.114 + \phi(1.1 + 43.1N^{1.25}) \quad \text{(S8)}$$

Where N is the model fraction of sucrose calculated and φ is a temperature function expressed by equations S9 and S10 respectively.

$$N = \frac{B}{1900 - 18B} \quad \text{(S9)}$$

$$\phi = \frac{30 - T}{91 - T} \quad \text{(S10)}$$

Where B is the °Brix and T is the temperature.

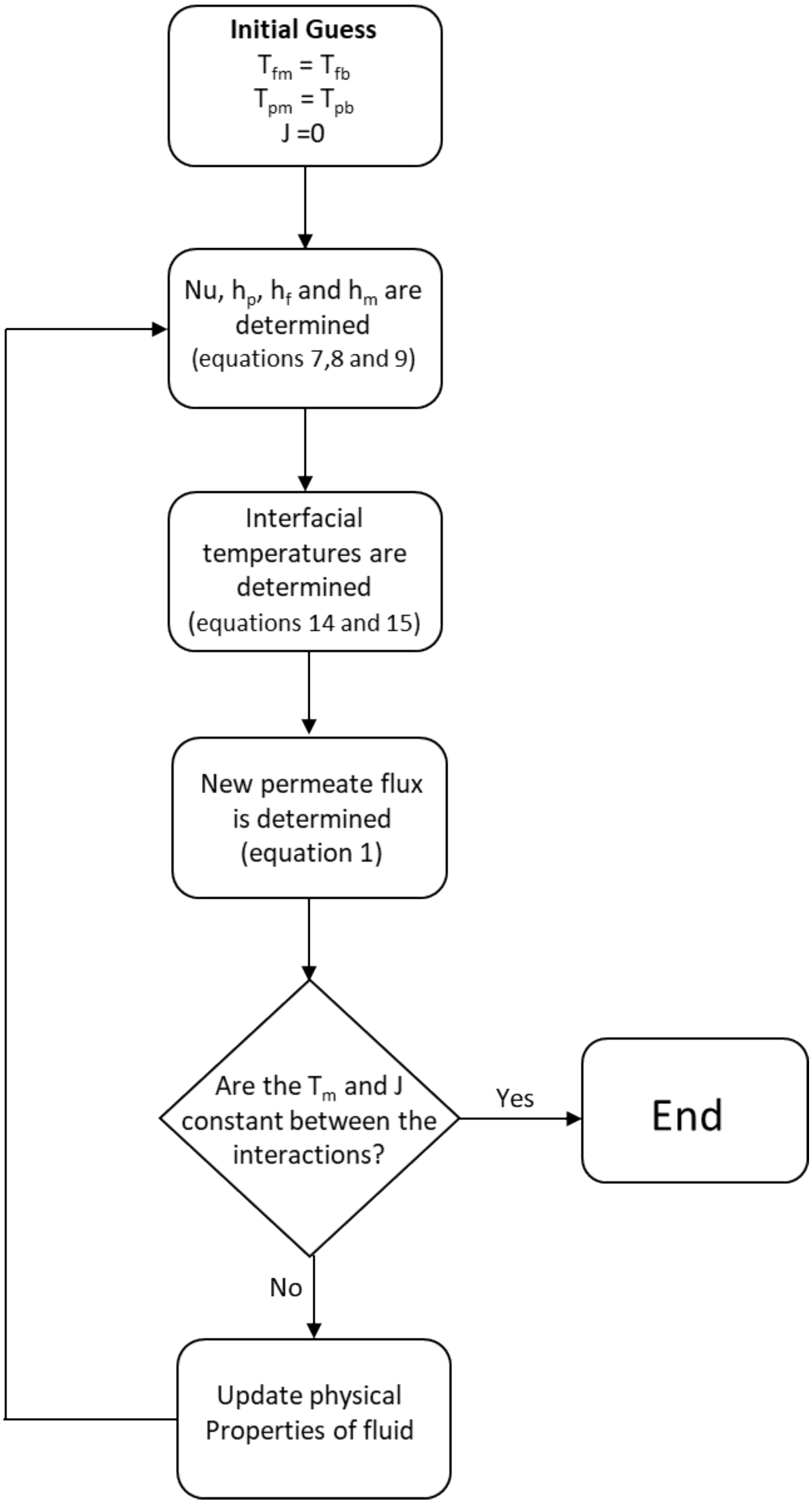

Figure S1: Algorithm for simulation of membrane distillation using Nusselt equation. Adapted by: [2]

Efficiency parameters of the membrane distillation process

MD can be considered an attractive technology in terms of energy efficiency because of its ability to apply low-temperature differences and sustainable or low-quality heat energy such as solar, waste heat, or geothermal energy [11]. These features may make it a process economically viable. The efficiency of membrane distillation process is usually evaluated by the gained output ratio (GOR) and specific energy consumption (SEC) [12]. GOR is a non-dimensional parameter and is defined as the ratio of the heat associated with the water vapor transfer to the total heat input, as shown in Equation S11 [13]. High GOR values indicate a lower thermal energy consumption per unit of permeate mass.

$$GOR = \frac{\dot{m}_p \Delta H_v}{\dot{Q}_{perm}} \qquad \text{(S11)}$$

Where is the $\dot{m}_p$ the permeate mass flow rate, ΔH_v is the specific enthalpy of evaporation and $\dot{Q}_{perm}$ is the power input in the heater.

When energy recovery is considered with efficient isolation or by the re circulation of the feed, the GOR achieves values from 1.5 to 10 [14], [15], [16], [17].

The SEC is defined as the ratio of the amount of energy consumed to the permeate mass unit [18], [19]as shown in Equation S12.

$$SEC = \frac{\dot{Q}_m \rho_w}{JA} \qquad \text{(S12)}$$

Where $\dot{Q}_m$ is the total heat flux through the membrane, ρ_w is the water density, J is the permeate flux and A is the membrane area.

Landfill gas generation (LFG) and on-site waste heat recovery

The methodology applied is based on a first-order degradation model, which includes two model parameters, L_0 and K, which represent the methane production potential and the first-order decay rate constant [20]. The first-order degradation model (LandGEN) was applied to estimate the amounts of landfill gas and methane produced by the MSW landfill. In the LandGEM formulation, methane generation can be expressed using Equation S13.

$$Q_{CH_4} = kL_0 \sum_{i=1}^{y} \sum_{j=0.1}^{1} \left(\frac{M_i}{10}\right) e^{-kt_{i,j}} \quad \text{(S13)}$$

Q_{CH_4} is the computed CH_4 gas generation for a specific year y (m^3 CH_4 yr^{-1}); k is the first-order decay rate constant (yr^{-1}), L_0 is the methane generation potential (m^3 CH_4 wet Mg^{-1}), M_i is the mass of waste landfilled in the ith year (Mg), i is the range from 1 to y, j is the intra-annual time increment used to calculate CH_4 generation, and t is the time in years.

Two default values exist for L_0 and K within the LandGEM approach: the clean air act (CAA) and AP-42. CAA defaults are required to predict that MSW landfills will require gas collection systems. In contrast, AP-42 default values are used for greenhouse gas emission inventories and to size the gas collection and control system based on the expected peak flow of landfill gas. The default LandGEM values are listed in Table S5. In this study, the CAA default was adopted.

Table S5: LanGEM default parameter. Source: [20]

Default	L_0 [m^3 of CH_4 Mg^{-1}]	k [yr^{-1}]
CAA	170	0.05[a]
		0.02[b]
AP-42	100	0.04[a]
		0.02[b]
	96[c]	0.7

[a]: Landfill receives > 63.5 cm precipitation/yr

[b]: Landfill receives <63.5 cm precipitation/yr

[c]: bioreactor

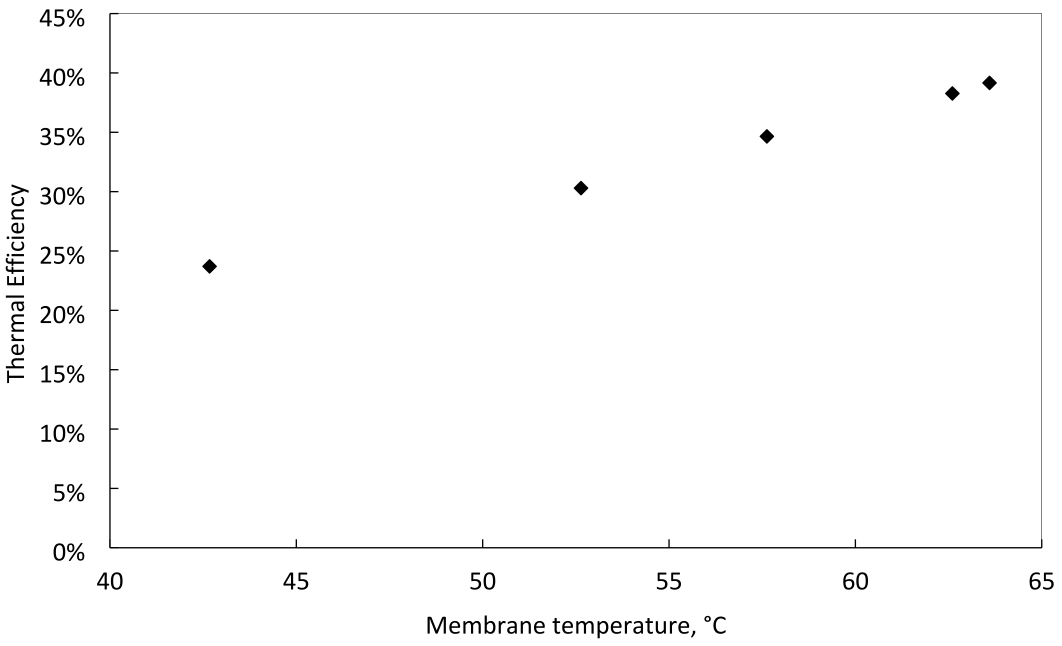

Figure S2: Effect of feed temperature on thermal efficiency of direct contact membrane distillation.

References

[1] A. Dan *et al.*, “Removal of heavy metals from synthetic landfill leachate in lab-scale vertical flow constructed wetlands,” *Science of the Total Environment*, vol. 584–585, pp. 742–750, 2017, doi: 10.1016/j.scitotenv.2017.01.112.

[2] I. Hitsov, T. Maere, K. De Sitter, C. Dotremont, and I. Nopens, “Modelling approaches in membrane distillation: A critical review,” *Sep Purif Technol*, vol. 142, pp. 48–64, Mar. 2015, doi: 10.1016/j.seppur.2014.12.026.

[3] J. Phattaranawik, R. Jiraratananon, and A. G. Fane, “Heat transport and membrane distillation coefficients in direct contact membrane distillation,” *J Memb Sci*, vol. 212, no. 1–2, pp. 177–193, Feb. 2003, doi: 10.1016/S0376-7388(02)00498-2.

[4] A.-S. Jönsson, R. Wimmerstedt, and A.-C. Harrysson, “Membrane distillation - a theoretical study of evaporation through microporous membranes,” *Desalination*, vol. 56, pp. 237–249, Jan. 1985, doi: 10.1016/0011-9164(85)85028-1.

[5] G. S. Manjunatha, D. Chavan, P. Lakshmikanthan, L. Singh, S. Kumar, and R. Kumar, “Specific heat and thermal conductivity of municipal solid waste and its effect on landfill fires,” *Waste Management*, vol. 116, pp. 120–130, Oct. 2020, doi: 10.1016/j.wasman.2020.07.033.

[6] J. M. Smith, H. C. Van Ness, and M. Abbott, *Introduction to Chemical Engineering Thermodynamics*. in CHEMICAL ENGINEERING SERIES. McGraw-Hill Education, 2005. [Online]. Available: https://books.google.com.br/books?id=c7J4TRxbGq8C

[7] R. W. Schofield, A. G. Fane, C. J. D. Fell, and R. Macoun, “Factors affecting flux in membrane distillation,” *Desalination*, vol. 77, pp. 279–294, Mar. 1990, doi: 10.1016/0011-9164(90)85030-E.

[8] P. Reiser, G. G. Birch, and M. Mathlouthi, “Physical properties,” in *Sucrose*, Boston, MA: Springer US, 1995, pp. 186–222. doi: 10.1007/978-1-4615-2676-6_8.

[9] A. Simion, C. G. Grigoras, L. Rusu, and A. Dabija, “Modeling of the thermo-physical properties of aqueous sucrose solutions ii. boiling point, specific heat capacity and thermal conductivity,” 2017. [Online]. Available: https://api.semanticscholar.org/CorpusID:106124657

[10] M. Mathlouthi and J. Génotelle, “Rheological properties of sucrose solutions and suspensions,” in *Sucrose*, Boston, MA: Springer US, 1995, pp. 126–154. doi: 10.1007/978-1-4615-2676-6_6.

[11] R. Ullah *et al.*, “Energy efficiency of direct contact membrane distillation,” *Desalination*, vol. 433, pp. 56–67, May 2018, doi: 10.1016/j.desal.2018.01.025.

[12] M. R. Elmarghany, A. H. El-Shazly, M. S. Salem, M. N. Sabry, and N. Nady, “Thermal analysis evaluation of direct contact membrane distillation system,” *Case Studies in Thermal Engineering*, vol. 13, p. 100377, Mar. 2019, doi: 10.1016/j.csite.2018.100377.

[13] J. Swaminathan, H. W. Chung, D. M. Warsinger, and J. H. Lienhard V, “Membrane distillation model based on heat exchanger theory and configuration comparison,” *Appl Energy*, vol. 184, pp. 491–505, Dec. 2016, doi: 10.1016/j.apenergy.2016.09.090.

[14] E. K. Summers, H. A. Arafat, and J. H. Lienhard, “Energy efficiency comparison of single-stage membrane distillation (MD) desalination cycles in different configurations,” *Desalination*, vol. 290, pp. 54–66, Mar. 2012, doi: 10.1016/j.desal.2012.01.004.

[15] G. Zuo, R. Wang, R. Field, and A. G. Fane, "Energy efficiency evaluation and economic analyses of direct contact membrane distillation system using Aspen Plus," *Desalination*, vol. 283, pp. 237–244, Dec. 2011, doi: 10.1016/j.desal.2011.04.048.

[16] Q. He, P. Li, H. Geng, C. Zhang, J. Wang, and H. Chang, "Modeling and optimization of air gap membrane distillation system for desalination," *Desalination*, vol. 354, pp. 68–75, Dec. 2014, doi: 10.1016/j.desal.2014.09.022.

[17] H. Geng, Q. He, H. Wu, P. Li, C. Zhang, and H. Chang, "Experimental study of hollow fiber AGMD modules with energy recovery for high saline water desalination," *Desalination*, vol. 344, pp. 55–63, Jul. 2014, doi: 10.1016/j.desal.2014.03.016.

[18] M. I. Soomro and W.-S. Kim, "Parabolic-trough plant integrated with direct-contact membrane distillation system: Concept, simulation, performance, and economic evaluation," *Solar Energy*, vol. 173, pp. 348–361, Oct. 2018, doi: 10.1016/j.solener.2018.07.086.

[19] G. Guan, X. Yang, R. Wang, and A. G. Fane, "Modular matrix design for large-scale membrane distillation system via Aspen simulations," *Desalination*, vol. 428, pp. 207–217, Feb. 2018, doi: 10.1016/j.desal.2017.11.033.

[20] W. Sun, X. Wang, J. F. DeCarolis, and M. A. Barlaz, "Evaluation of optimal model parameters for prediction of methane generation from selected U.S. landfills," *Waste Management*, vol. 91, pp. 120–127, May 2019, doi: 10.1016/j.wasman.2019.05.004.
